# Transcriptomics Study on *Staphylococcus aureus* Biofilm Under Low Concentration of Ampicillin

**DOI:** 10.3389/fmicb.2018.02413

**Published:** 2018-10-30

**Authors:** Junyan Liu, Ling Yang, Yuchao Hou, Thanapop Soteyome, Bingbing Zeng, Jianyu Su, Lin Li, Bing Li, Dingqiang Chen, Yanyan Li, Aiwu Wu, Mark E. Shirtliff, Janette M. Harro, Zhenbo Xu, Brian M. Peters

**Affiliations:** ^1^School of Food Science and Engineering, South China University of Technology, Guangzhou, China; ^2^Department of Clinical Pharmacy, College of Pharmacy, University of Tennessee Health Science Center, Memphis, TN, United States; ^3^Department of Laboratory Medicine, The First Affiliated Hospital of Guangzhou Medical University, Guangzhou Medical University, Guangzhou, China; ^4^Home Economics Technology, Rajamangala University of Technology Phra Nakhon, Bangkok, Thailand; ^5^Zhuhai Encode Medical Engineering Co., Ltd., Zhuhai, China; ^6^Guangdong Province Key Laboratory for Green Processing of Natural Products and Product Safety, Guangzhou, China; ^7^Department of Laboratory Medicine, Zhujiang Hospital, Southern Medical University, Guangzhou, China; ^8^Department of Cell Biology, Harvard Medical School, Boston, MA, United States; ^9^KingMed School of Laboratory Medicine, Guangzhou Medical University, Guangzhou, China; ^10^Department of Microbial Pathogenesis, School of Dentistry, University of Maryland, Baltimore, MD, United States; ^11^Overseas Expertise Introduction Center for Discipline Innovation of Food Nutrition and Human Health (111 Center), Guangzhou, China

**Keywords:** transcriptomics, RNA-sequencing, *Staphylococcus aureus*, biofilm formation, ampicillin

## Abstract

*Staphylococcus aureus* is one of the representative foodborne pathogens which forms biofilm. Antibiotics are widely applied in livestock husbandry to maintain animal health and productivity, thus contribute to the dissemination of antimicrobial resistant livestock and human pathogens, and pose a significant public health threat. Effect of antibiotic pressure on *S. aureus* biofilm formation, as well as the mechanism, remains unclear. In this study, the regulatory mechanism of low concentration of ampicillin on *S. aureus* biofilm formation was elucidated. The viability and biomass of biofilm with and without 1/4 MIC ampicillin treatment for 8 h were determined by XTT and crystal violet straining assays, respectively. Transcriptomics analysis on ampicillin-induced and non-ampicillin-induced biofilms were performed by RNA-sequencing, differentially expressed genes identification and annotation, GO functional and KEGG pathway enrichment. The viability and biomass of ampicillin-induced biofilm showed dramatical increase compared to the non-ampicillin-induced biofilm. A total of 530 differentially expressed genes (DEGs) with 167 and 363 genes showing up- and down-regulation, respectively, were obtained. Upon GO functional enrichment, 183, 252, and 21 specific GO terms in biological process, molecular function and cellular component were identified, respectively. Eight KEGG pathways including “Microbial metabolism in diverse environments”, “*S. aureus* infection”, and “Monobactam biosynthesis” were significantly enriched. In addition, “beta-lactam resistance” pathway was also highly enriched. In ampicillin-induced biofilm, the significant up-regulation of genes encoding multidrug resistance efflux pump AbcA, penicillin binding proteins PBP1, PBP1a/2, and PBP3, and antimicrobial resistance proteins VraF, VraG, Dlt, and Aur indicated the positive response of *S. aureus* to ampicillin. The up-regulation of genes encoding surface proteins ClfB, IsdA, and SasG and genes (*cap5B* and *cap5C*) which promote the adhesion of *S. aureus* in ampicillin induced biofilm might explain the enhanced biofilm viability and biomass.

## Introduction

Animal protein demand for human consumption is rising worldwide. Daily animal protein intake in Asia increased from 7 grams/capita/day in 1960 to 25 grams/capita/day in 2013^1^ (Guo et al., 2000). To meet the requirement, antibiotics are widely applied in livestock husbandry to maintain animal health and productivity, thus contribute to the dissemination of antimicrobial resistant livestock and human pathogens, and pose significant public health implications (Van Boeckel et al., 2015). The average annual consumption of antibiotics worldwide was estimated to be 172 mg/kg, 148 mg/kg, and 45 mg/kg for pigs, chicken, and cattle, respectively (Van Boeckel et al., 2015). In the United States, antimicrobial use in food animals is estimated to account for approximately 80% of the nation’s annual antimicrobial consumption (Food and Drug Administration, 2010). In 2010, China was the largest antimicrobial consumer for livestock and the consumption of antibiotics in China was estimated at 162,000 tons in 2013, with 52% applied in animals (Van Boeckel et al., 2015; Zhang et al., 2015). This widespread use of antibiotics in livestock contributes to the emergence of antimicrobial resistant pathogenic bacteria and lead to significant public health threats. Repeated exposure to low doses of antimicrobial agents builds optimum conditions for the emergence and dissemination of antimicrobial resistant bacteria in animals (You and Silbergeld, 2014; Lin et al., 2018). Animal origin antimicrobial resistant bacteria can be transmitted to agricultural workers by direct contact and to other humans through food products and environment (Price et al., 2005; Graham et al., 2009; Smith et al., 2013; Xu et al., 2017).

*Staphylococcus aureus* is one of the most common human and animal pathogens and one of the first strains characterized to be resistant to antimicrobials. Penicillin was first used in animal production in the late 1940s, and *S. aureus* with resistance to penicillin were observed in 1948 (Huttner et al., 2013). Nowadays, most of the *S. aureus* strains show resistance to penicillin by producing beta-lactams. In the past decade, *S. aureus* biofilms which are major cause for concern in multiple infections and are associated with chronic infections, have brought increased recognition (Conlon, 2014). Both *in vitro* and *in vivo* studies have showed biofilms pose challenges to medical and industrial due to their increased tolerance of antimicrobials and disinfectants (Bjarnsholt et al., 2013; Neopane et al., 2018). Biofilm development was classified into three stages: initial attachment, biofilm maturation, and dispersal and later proposed to include five stages: attachment, multiplication, exodus, maturation, and dispersal (O’Toole et al., 2000; Moormeier et al., 2014; Miao et al., 2017). Biofilm growth plays a major role during bacterial infection by the defense against several clearance mechanisms (Stewart and Costerton, 2001). Biofilm formation of *S. aureus* is also linked with irons, virulence factors, surface proteins and accessory gene regulator (agr) quorum-sensing system whose expression depends on environmental conditions (Cucarella et al., 2001; Yarwood et al., 2004; Poupel et al., 2018; Swarupa et al., 2018). In addition, biofilm cells display enhanced resistance to antibiotics (de la Fuente-Nunez et al., 2013; Lin et al., 2018). It has been reported that *S. aureus* isolated from hospitalized patients have high degree of biofilm-forming ability with high tendency to exhibit antimicrobial resistance, multidrug resistance and methicillin resistance (Neopane et al., 2018). Subinhibitory concentration of methicillin has been shown to lead to dramatic increase in biofilm formation of *S. aureus* which was dependent on autolysis activity linked to *atl* (Kaplan et al., 2012; Ranieri et al., 2018).

However, the effect of low-concentration of ampicillin pressure appeared in food products on *S. aureus* biofilm formation, as well as the mechanism, remains unclear. In this study, the regulatory mechanism of low concentration of ampicillin as a common antibiotic applied in livestock husbandry on *S. aureus* biofilm formation was elucidated by transcriptomics analysis.

## Materials and Methods

### Bacterial Strains and Growth Conditions

*Staphylococcus aureus* strain FAHGMU10071 was isolated from a patient in the First Affiliated Hospital of Guangzhou Medical University in Guangzhou, China and was maintained as a glycerol stock stored at -80°C. A small amount of stock was spread onto Tryptone Soy agar (TSA) and incubated at 37°C for 24 h to obtain isolated colonies. A single colony was transferred to 2 mL of Tryptone Soy broth (TSB) and incubated at 37°C with shaking at 200 rpm overnight prior to further experiments. The minimal inhibitory concentration (MIC) was measured by broth microdilution method.

### Biofilm Sample Collection

40 mL of overnight *S. aureus* culture was inoculated into 2 mL fresh TSB and incubated at 37°C with shaking at 200 rpm for 3 h to reach logarithmic growth phase. The logarithmic growth culture was then washed twice in sterile PBS and diluted to 10^7^ cells/mL in TSB. 5 mL of the culture was added into a flat-bottomed 6-well plate (Trasadingen, Switzerland) and incubated at 37°C with gentle shaking (10–20 rpm) for 8 h to form biofilm. The biofilm viability and biomass were determined by XTT and Crystal Violet Staining assays, respectively (Christensen et al., 1985; Silva et al., 2008). The experiment was performed in triplicate and biofilm samples were collected at 8 h to be control groups (C1, C2, and C3) For ampicillin-induced groups (A1, A2, and A3), 1/4 MIC of ampicillin was added into the flat-bottomed 6-well plate at the meantime and biofilm samples were collected at 8 h.

### RNA Isolation, Library Construction and Sequencing

Total RNA of *S. aureus* biofilm samples (A1, A2, A3, C1, C2, and C3) were extracted using the TRizol reagent (Sigma-Aldrich, United States) and Bacterial Total RNA Extraction kit (Sigma-Aldrich, United States) based on the manufacturer’s instruction (Liu et al., 2016a,b). The quality and quantity of the RNA samples were determined by Ultramicro spectrophotometer K5500 (Beijing Kaiao Technology Development Co., Ltd., China) and Agilent 2200 TapStation (Agilent Technologies, United States). To remove trace DNA and ribosomal RNAs, RNase-free DNase I (Ambion Inc., United States) and MICROBExpressTM kit (Ambion Inc., United States) were applied, respectively (Liu et al., 2016a). The mRNA was fragmented ultrasonically and was then reverse transcribed to cDNA (Liu et al., 2016a). RNA-seq libraries construction and RNA sequencing were performed in biological triplicate by RIBOBIO Biotechnology Company (Guangzhou, China) using the Illumina Paired End Sample Prep kit and the Illumina Hiseq 2500 platform, respectively (Liu et al., 2016a).

### Differentially Expressed Genes Identification and Annotation

Raw reads were generated from image data and stored as FASTQ format. Raw data were filtered to remove adaptor contaminated and low quality sequences and obtain clean reads. Clean reads were quality examined by FastQC v.0.10.1^2^ and aligned to reference genome of *S. aureus* NCTC 8325 (NCBI Reference Sequence: NC_007795) using TopHat (Trapnell et al., 2009). No more than 2 bases mismatch and read gap were allowed in the alignment. Gene coverage was calculated by the percentage of genes covered by reads and gene functional annotation was performed through ANNOVAR (Wang et al., 2010). Differentially expressed genes (DEGs) were identified using DEGseq panormalage (Anders, 2010; Anders and Huber, 2012) based on negative binomial distributions. The gene expression level was measured based on Reads Per Kilobase of transcript per Million mapped reads (RPKM) values and *P* values adjusted using the edgeR panormalage (Mortazavi et al., 2008; Robinson et al., 2010). Genes with an adjusted |log_2_(fold change)| > 1 and *P*-value < 0.05 were identified as DEGs. Kyoto encyclopedia of genes and genomes (KEGG) pathway and Gene Ontology (GO) annotation of DEGs were performed by kobas based on KEGG database (Kanehisa and Goto, 2000) and through GO database (Ashburner et al., 2000), respectively. The significantly enriched KEGG pathways and GO terms were identified by *P*-value < 0.05 in Fisher Exact Test and *P*-value < 0.01 in Hypergeometric Distribution, respectively, and adjusted by false discovery rates (FDR) (Storey, 2002; Rivals et al., 2007).

### RT-PCR Validation

In order to validate the RNA-seq data, RT-PCR was performed to quantify the mRNA transcripts of 10 selected DEGs using the Light Cylcler 480 (Roche, Switzerland) according to the manufacturer’s instructions. Each RT-PCR reaction was performed in a final volume of 25 μL. The thermal cycling profile was as follows: 42°C for 60 min and 72°C for 10 min; 40 cycles of 95°C for 15 s, 60°C for 30 s, 72°C for 30 s, and a final extension of 68°C for 10 min. Negative control samples containing sterile water were also included. The cycle threshold values (C_T_) were determined and the relative fold differences were calculated by the 2^-ΔΔCT^ method^54^ using *16S rRNA* as the reference gene. Three independent experiments were run in triplicate.

## Results and Discussion

### General Features of the Transcriptome Profile

**FIGURE 1:**
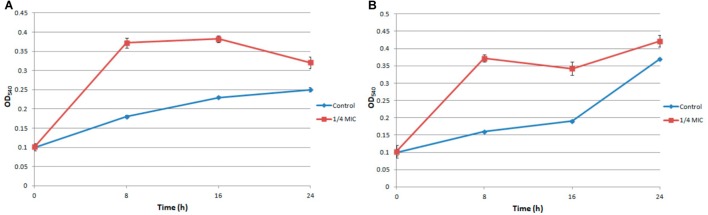
The biomass **(A)** and viability **(B)** of ampicillin induced *S. aureus* biofilm.

Under the treatment of 1/4 MIC ampicillin for 8 h, the biofilm of *S. aureus* strain FAHGMU10071 showed dramatical increase in biomass and viability compared to the no treatment control (Figure 1; Xu et al., 2018). To gain further insight into the regulatory mechanism of low concentration antibiotic on *S. aureus* biofilm formation, RNA-seq analyses were performed on three biological replicates of *S. aureus* biofilm grown at 37°C for 8 h with (A1, A2, and A3) and without (C1, C2, and C3) 1/4 MIC of ampicillin. The cDNA libraries of the 6 *S. aureus* biofilm samples were constructed, sequenced and generated with a total of 29,684,588 to 40,756,932 reads which were mapped to the reference genome of *S. aureus* NCTC 8325 (NCBI Reference Sequence: NC_007795) (Supplementary Table S1). The overall gene expression levels of the three biological replicates of each group showed high similarity with each other, illustrating that the RNA-seq data were available for transcriptome analysis (NCBI SRA accession number: SRP154796). Gene expression levels determined by the average RPKM values demonstrated that 2840 total genes were expressed in ampicillin-induced and control groups.

### Differentially Expressed Gene (DEG)

**FIGURE 2:**
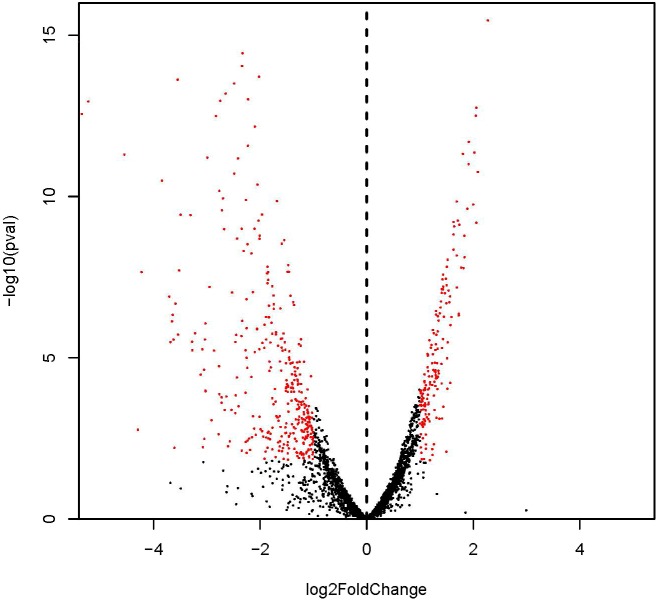
Differential expression level of ampicillin-induced (A1, A2, and A3) and control (C1, C2, and C3) groups identified by | log_2_(fold change)| > 1 and adjust *P*-value < 0.05.

Identified by adjusted |log_2_(fold change)| > 1 and *P*-value < 0.05 (Figure 2; Tan et al., 2015), 530 DEGs with 167 and 363 genes showing up- and down-regulation, respectively, were obtained (Supplementary Figure S1 and Supplementary Table S2). Considering the most differentially expressed genes, 6 of the significantly up-regulated genes showed |log_2_(fold change)| > 2 and 10 of the significantly down-regulated genes showed |log_2_(fold change)| > 4. The 6 up-regulated genes encode cation transporter E1-E2 family ATPase, argininosuccinate synthase, 6-phospho-beta-galactosidase, argininosuccinate lyase, PTS system lactose-specific transporter subunit IIBC, and riboflavin biosynthesis protein, respectively. The 10 down-regulated genes encode carbamate kinase, arginine/ornithine antiporter, 2-isopropylmalate synthase, ornithine carbamoyltransferase, phage head protein, 23S Ribosomal RNA, and 4 hypothetical proteins, respectively.

Considering the *S. aureus* biofilm formation, DEGs in ampicillin-induced biofilm include some key genes encoding surface proteins, virulence factors, adhesins, proteases, and capsular polysaccharide proteins (Wang and Wood, 2011). Genes *cap5B* and *cap5C* mediating the capsular polysaccharide biosynthesis were significantly up-regulated. The *S. sureus* strain FAHGMU10071 might promote biofilm formation by the synthesis of capsular polysaccharides under low concentration of ampicillin. However, the expressions of genes encoding proteases ClpB and ClpC which are required for stress tolerance, intracellular replication and biofilm formation (Frees et al., 2004) and genes encoding adhesins SdrD and Fib significantly decreased. A newly reported virulence factor, spdC, which controls histidine kinase activity and the master regulator of cysteine metabolism, cymR, which plays a role in biofilm formation in *S. aureus* showed no significantly differential expression (Soutourina et al., 2009; Poupel et al., 2018).

### GO Functional Enrichment Analysis

**FIGURE 3:**
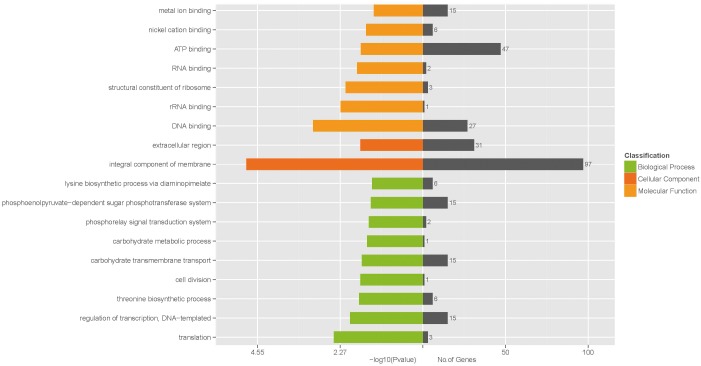
Significantly enriched GO terms of differentially expressed genes.

To further understand the function of the DEGs underlying the effect of low concentration of ampicillin on *S. aureus* biofilm, GO enrichment analysis was performed with the 530 DEGs. Based on sequence homology, DEGs were assigned to one or more GO terms and categorized into 535, 536, and 277 secondary level GO terms in the three main categories (biological process, molecular function, and cellular component) of the GO function, respectively. Upon GO functional enrichment, 183, 252, and 21 specific GO terms in biological process, molecular function and cellular component were identified, respectively (Supplementary Table S3). Significantly enriched GO terms composed of the three specific categories were illustrated in Figure 3. Nine GO terms were enriched in the category of biological process, including “translation,” “regulation of transcription, DNA-templated,” “threonine biosynthetic process,” “cell division,” “carbohydrate transmembrane transport,” “carbohydrate metabolic process,” “phosphorelay signal transduction system,” “phosphoenolpyruvate-dependent sugar phosphotransferase system,” and “lysine biosynthetic process via diaminopimelate”. In the category of molecular function, “DNA binding,” “rRNA binding,” “structural constituent of ribosome,” “RNA binding,” “ATP binding,” “nickel cation binding,” and “metal ion binding” were significantly enriched. Only 2 GO terms “integral component of membrane” and “extracellular region” were enriched in the category of cellular component.

### KEGG Pathway Enrichment

**FIGURE 4:**
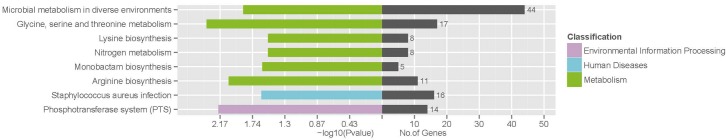
Significantly enriched KEGG pathways of differentially expressed genes.

To identify the involved pathways, DEGs were mapped to the KEGG database, followed by KEGG pathway enrichment analysis (Supplementary Table S4). KEGG pathways “Glycine, serine and threonine metabolism,” “Phosphotransferase system (PTS),” “Arginine biosynthesis,” “Microbial metabolism in diverse environments,” “*Staphylococcus aureus* infection,” “Monobactam biosynthesis,” “Nitrogen metabolism,” and “Lysine biosynthesis” were significantly enriched (Figure 4).

**FIGURE 5:**
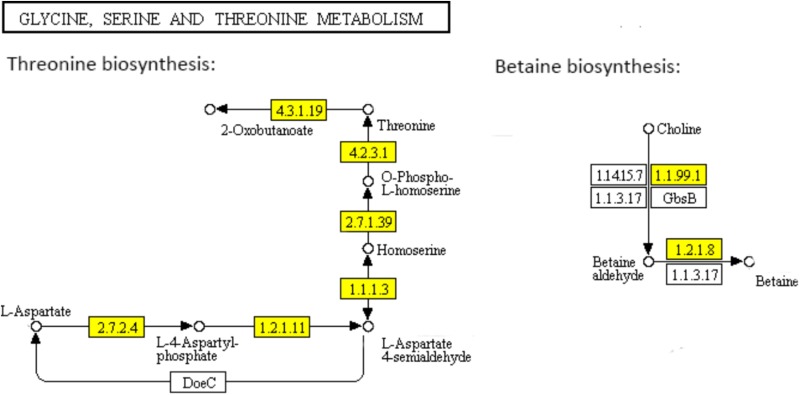
Significantly enriched KEGG pathway “Glycine, serine and threonine metabolism” (Yellow highlights represent down-regulation and green highlights represent up-regulation) (Kanehisa and Goto, 2000; Kanehisa et al., 2017).

Threonine which is an essential amino acid cannot be synthesized by animals, is derived from aspartate in bacteria and plants. In the “Glycine, serine and threonine metabolism” pathway (Figure 5), aspartate kinase (EC: 2.7.2.4), aspartate semialdehyde dehydrogenase (EC: 1.2.1.11), homoserine dehydrogenase (EC: 1.1.1.3), homoserine kinase (EC: 2.7.1.39), and threonine synthase (EC: 4.2.3.1) are functional during the threonine biosynthesis (aspartate - homoserine - threonine) (Rees and Hay, 1995; Gaupp et al., 2010). L-aspartate is phosphorylated by aspartate kinase to produce L-4-aspartylphosphate, which is reduced to L-aspartate-4-semialdehyde by aspartate semialdehyde dehydrogenase. The semialdehyde is further reduced by homoserine dehydrogenase to the non-protein amino acid homoserine. Homoserine kinase phosphorylates homoserine to produce O-phospho-L-homoserine, which is converted to threonine by threonine synthase (Rees and Hay, 1995). All the genes encoding the key functional enzymes in the threonine biosynthesis process showed significant down-regulation in the ampicillin-induced biofilm, indicating the attenuated activation of threonine biosynthesis pathway during the formation of *S. aureus* biofilm with antibiotic treatment. The functional enzymes including choline dehydrogenase (EC: 1.1.99.1) and betaine aldehyde dehydrogenase (EC: 1.2.1.8) during the betaine biosynthesis (choline - betaine) in the “Glycine, serine and threonine metabolism” pathway (Figure 5) were also significantly down-regulated in the ampicillin-induced biofilm.

**FIGURE 6:**
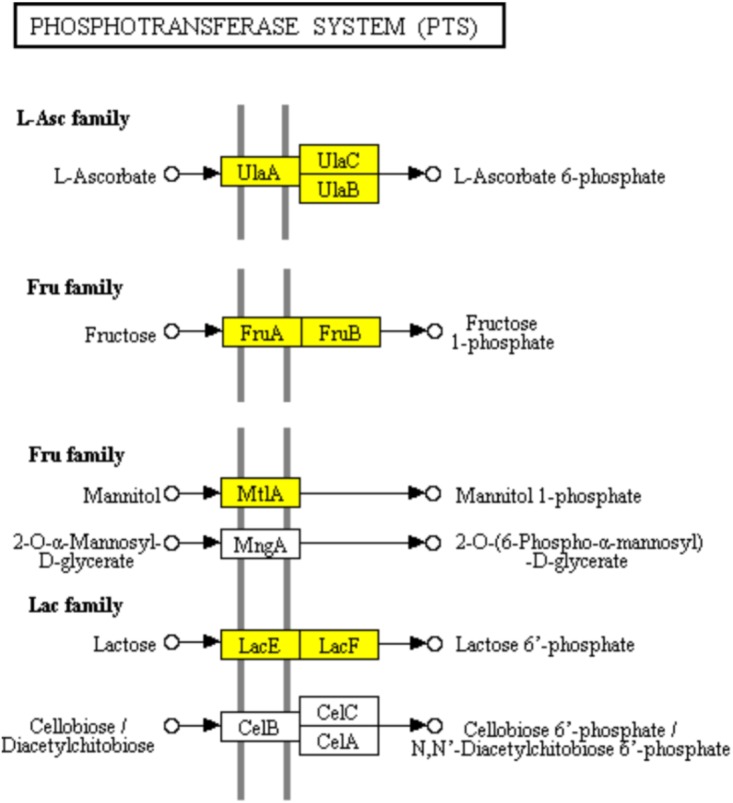
Significantly enriched KEGG pathway “Phosphotransferase system (PTS)” (Yellow highlights represent down-regulation and green highlights represent up-regulation) (Kanehisa and Goto, 2000; Kanehisa et al., 2017).

The PTS has been characterized as a major bacterial mechanism for uptake of carbohydrates, especially hexoses, hexitols, and disaccharides, which are referred to PTS carbohydrate (Kotrba et al., 2001). The uptake of carbohydrates and their conversion are catalyzed into the respective phosphoesters by PTS during transport process (Tchieu et al., 2001). Four successive phosphoryl transfers appear in the PTS (Tchieu et al., 2001). Using phosphoenolpyruvate (PEP) as a substrate, initial autophosphorylation of enzyme I (EI) is followed by transfer of the phosphoryl group from EI to histidine phosphocarrier protein (HPr) (Tchieu et al., 2001). The self-phosphoryl transfer from HPr, after which the phosphoryl group is transferred to histidine or cysteine residues of EIIB, are catalyzed by enzyme II (EII) A (Tchieu et al., 2001). The specific saccharide is transported through the membrane-bound EIIC and is phosphorylated by the appropriate saccharide-specific EIIB (Tchieu et al., 2001). In the L-ascorbate family, the genes encoding EIIABC (UlaA, UlaB, and UlaC) which catalyzed, transported, and phosphorylated L-ascorbate to L-arsorbate 6-phosphate, were significantly down-regulated. It suggested the lower uptake of L-ascorbate in ampicillin-induced biofilm (Figure 6). The up-regulation of genes encoding EIIABC in the Lactose family (LacE and LacF) and Fructose family (MtlA, FruA and FruB) revealed the diminishing uptake of lactose and fructose in ampicillin-induced biofilm (Figure 6).

**FIGURE 7:**
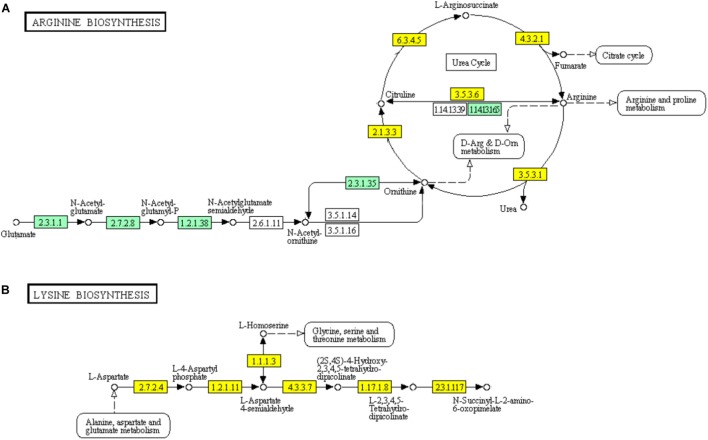
Significantly enriched KEGG pathways “Arginine biosynthesis” **(A)** and “Lysine biosynthesis” **(B)** (Yellow highlights represent down-regulation and green highlights represent up-regulation) (Kanehisa and Goto, 2000; Kanehisa et al., 2017).

In the urea cycle of “Arginine biosynthesis” pathway, genes encoding major enzymes including ornithine carbamoyltransferase (EC: 2.1.3.3), argininosuccinate synthase (EC: 6.3.4.5), argininosuccinate lyase (EC: 4.3.2.1), arginase (EC: 3.5.3.1), and arginine deiminase (EC: 3.5.3.6) showed significant down-regulation (Figure 7). However, the genes encoding bifunctional ornithine acetyltransferase/N-acetylglutamate synthase argJ (EC: 2.3.1.1) acetylglutamate kinase (EC: 1.2.1.38), and N-acetyl-gamma-glutamyl-phosphate reductase argC (EC: 2.7.2.8) which catalyze glutamate to N-acetylglutamate semialdehyde were significantly up-regulated (Figure 7A). The diaminopimelic acid pathway (DAP) in “Lysine biosynthesis” pathway has been reported to be related to arginine metabolism (Velasco et al., 2002). Similar to the genes involved in “Arginine biosynthesis” pathway, the genes encoding enzymes [homoserine dehydrogenase (EC: 1.1.1.3), aspartate kinase (EC: 2.7.2.4, EC: 2.7.2.4), aspartate semialdehyde dehydrogenase (EC: 1.2.1.11), 4-hydroxy-tetrahydrodipicolinate synthase (EC: 4.3.3.7), and 4-hydroxy-tetrahydrodipicolinate reductase (EC: 1.17.1.8)] involved in DAP were significantly down-regulated (Figure 7B).

**FIGURE 8:**
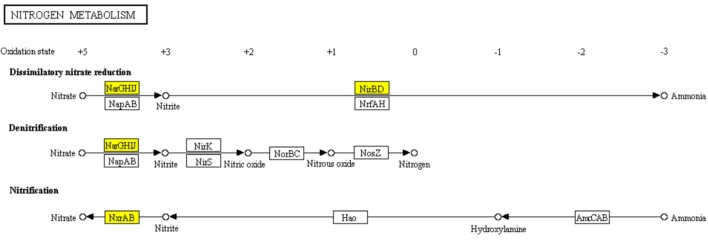
Significantly enriched KEGG pathway “Nitrogen metabolism” (Yellow highlights represent down-regulation and green highlights represent up-regulation) (Kanehisa and Goto, 2000; Kanehisa et al., 2017).

The core nitrogen cycle in “Nitrogen metabolism” pathway involves four reduction pathways and two oxidation pathways. In ampicillin induced *S. aureus* biofilm, 6 genes encoding nitrate reductase NarGHIJ, NirBD, and NxrAB involved in the dissimilatory nitrate reduction, denitrification, and nitrification were significantly down-regulated (Figure 8). It has been reported that nitrogen metabolism is involved in antibiotic resistance (Borriello et al., 2004; Yan et al., 2013). Thus, the significant enrichment of “Nitrogen metabolism” pathway in ampiciliin induced biofilm group indicated the response of *S. aureus* biofilm to ampicillin. Besides, a total of 44 genes involved in “Microbial metabolism in diverse environments” were significantly up- or down-regulated, demonstrating the response of *S. aureus* biofilm to ampicillin.

### DEGs Involved in “*Staphylococcus aureus* infection” Pathway

**FIGURE 9:**
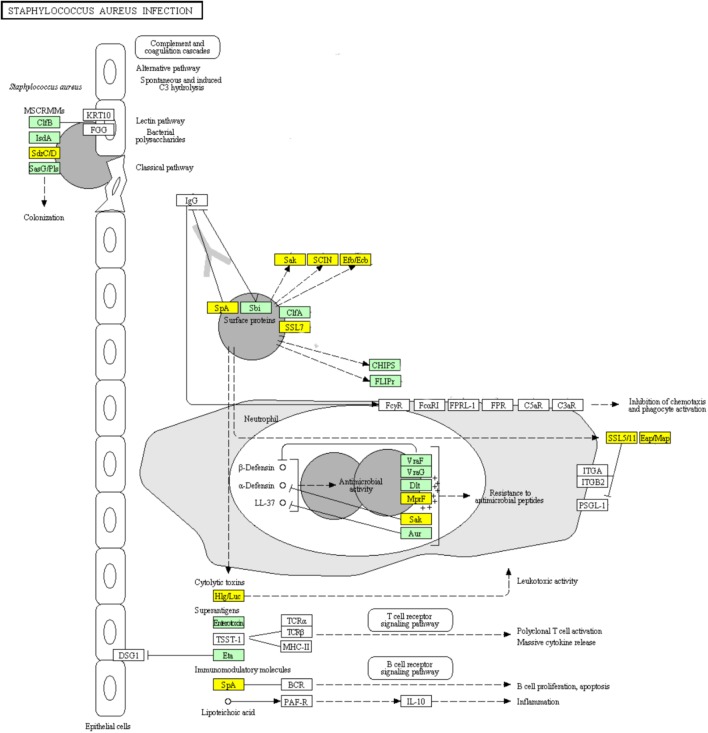
Significantly enriched KEGG pathway “*Staphylococcus aureus* infection” (Yellow highlights represent down-regulation and green highlights represent up-regulation) (Kanehisa and Goto, 2000; Kanehisa et al., 2017).

Infections including superficial skin infections, food poisoning infections, and life-threatening infections can be caused by *S. aureus*, which diverts the effectiveness of the immune system in several ways: modulating the cationic antimicrobial peptides sensitivity by increasing the cytoplasmic membrane positive net charge, superantigens expression which prevent common immune response development or cause emetic response when ingested, and secreting immune modulating proteins which inhibit complement activation and neutrophil chemotaxis or lysis (Foster, 2009; Kanehisa et al., 2017). The ability of *S. aureus* to colonize the host and to form biofilm is likely to be partially determined by its adhesion ability (Foulston et al., 2014; Scherr et al., 2014). Surface proteins including clumping factor B (ClfB), iron-regulated surface determinant (IsdA), SdrC, SdrD, and *S. aureus* surface protein (SasG) have been reported to be able to promote adhesion *in vitro* (Foster, 2009). SasG-expressing strains of *S. aureus* were reported to form biofilm independently of the polysaccharide intercellular adhesin (PIA) (Corrigan et al., 2007). SasG is able to mask *S. aureus* microbial surface components recognizing adhesive matrix molecules (MSCRAMMs) binding to their ligands and to improve biofilm formation (Corrigan et al., 2007). The up-regulation of genes encoding surface proteins ClfB, IsdA, and SasG which promote the adhesion of *S. aureus* in ampicillin induced biofilm explained the enhanced biofilm viability and biomass (Figure 9). Clumping factor A (ClfA) which is the major fibrinogen-binding protein on the surface of cells from the stationary phase of growth, has been reported to act as a protective antigen and a virulence determinant (Josefsson et al., 2001). The up-regulation of *clfA* gene implied the protection of *S. aureus* encountering antibiotic stress by ClfA (Figure 9). Obviously, the significantly up-regulated genes encoding VraF, VraG, Dlt, Aur proteins sharing antimicrobial resistance activities indicated the positively response of *S. aureus* to ampicillin (Figure 9; Foster, 2009).

Concerning the toxins involved in this pathway, genes encoding cytolytic toxins Hlg/Luc and immunomodulatory module Spa showed significant down-regulation, while genes encoding superantigens enterotoxin and exfoliative toxin Eta were significantly up-regulated during ampicillin-induced biofilm formation (Figure 9). The genes encoding superantigenic toxic shock syndrome toxin-1 (TSST-1) did not show significantly differential expression.

### DEGs Involved in “Monobactam Biosynthesis” and “Beta-Lactam Resistance” Pathways

**FIGURE 10:**
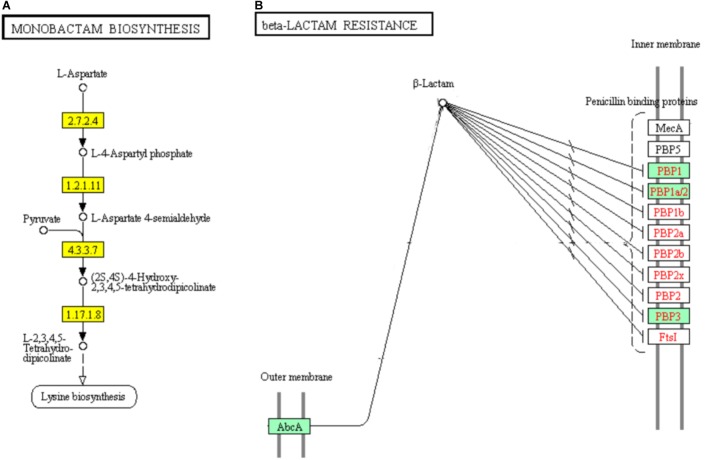
Significantly enriched KEGG pathways “Monobactam biosynthesis” **(A)** and “beta-lactam resistance” **(B)** (Yellow highlights represent down-regulation and green highlights represent up-regulation) (Kanehisa and Goto, 2000; Kanehisa et al., 2017).

Monobactams are beta-lactam antibiotics containing a monocyclic beta-lactam nucleus, that is different from penicillin and cephalosporin core structures with another fused ring (Hamed et al., 2013). The genes encoding aspartate kinase (EC: 2.7.2.4), aspartate semialdehyde dehydrogenase (EC: 1.2.1.11), 4-hydroxy-tetrahydrodipicolinate synthase (EC: 4.3.3.7), and 4-hydroxy-tetrahydrodipicolinate reductase (EC:1.17.1.8) which involved in part of the “Monobactam biosynthesis” pathway showed significant down-regulation (Figure 10A).

In addition, 5 of the 9 DEGs were involved in “beta-lactam resistance” pathway and showed significantly up-regulated in ampicillin-induced group compared to control group (Figure 10B). The up-regulation of “beta-lactam resistance” related genes indicated the strain was response to beta-lactam (ampicillin) stress. The beta-lactam antibiotics expand their effect in bacterial cell walls by interfering with the structural crosslinking of peptidoglycans. Bacterial beta-lactam antibiotic resistance can be acquired by strategies including the production of inactivated beta-lactamases, changing the beta-lactam targets for penicillin-binding proteins (PBPs), reducing the transport of beta-lactams into the periplasmic space through porin changes, and exclusion of beta-lactams via efflux pumps (Zapun et al., 2008; Kanehisa et al., 2017). In ampicillin-induced biofilm, genes encoding multidrug resistance efflux pump AbcA and penicillin binding proteins PBP1, PBP1a/2, and PBP3 were significantly up-regulated. The low concentration ampicillin resistance mechanism of *S. aureus* strain FAHGMU10071 might be the activation of multidrug resistance efflux pump AbcA and penicillin binding proteins PBP1, PBP2, and PBP3. PBPs which are enzymes involved in the last stages of peptidoglycan biosynthesis, have been intense studied especially considering the role of some important pathogens including *S. sureus* in the beta-lactams resistance. Four native PBPs (PBP1 to PBP4) and an methicillin-resistant *S. aureus* (MRSA) specific PBP2a have been identified in *S. aureus*. PBPs 1, 2 and 3 of *S. aureus* are essential for viability and are the targets of beta-lactam antibiotics (Waxman and Strominger, 1983). PBP1 of *S. aureus* has also been reported to be essential for growth and cell division (Pereira et al., 2007).

### RT-PCR Validation

Ten genes with different expression profiles were selected to validate the results of RNA-seq. The 10 genes were selected based on three criteria: (i) Gene function. The 10 genes were DEGs from different significantly enriched GO terms, COG categories and KEGG pathways. (ii) Expression level. Among the 10 genes, 5 were up-regulated and the others down-regulated. (iii) Gene position in the genome. The selected 10 genes were located at different positions in the genome. The mRNA levels of the 10 selected genes determined by qRT-PCR were consistent with those from the RNA-Seq analysis.

## Conclusion

In this study, the regulatory mechanism of low concentration of ampicillin on *S. aureus* biofilm formation was elucidated. The viability and biomass of ampicillin-induced biofilm showed dramatical increase compared to the non-ampicillin-induced biofilm. A total of 530 DEGs with 167 and 363 genes showing up- and down-regulation, respectively, were obtained by RNA-sequencing. Upon GO functional enrichment, 183, 252, and 21 specific GO terms in biological process, molecular function and cellular component were identified, respectively. Eight KEGG pathways including “Microbial metabolism in diverse environments,” “*Staphylococcus aureus* infection,” and “Monobactam biosynthesis” were significantly enriched. In addition, “beta-lactam resistance” pathway was also highly enriched. In ampicillin-induced biofilm, the significant up-regulation of genes encoding multidrug resistance efflux pump AbcA, penicillin binding proteins PBP1, PBP1a/2, and PBP3, and antimicrobial resistance proteins VraF, VraG, Dlt, and Aur indicated the positively response of *S. aureus* to ampicillin. The up-regulation of genes encoding surface proteins ClfB, IsdA, and SasG and genes (*cap5B* and *cap5C*) which promote the adhesion of *S. aureus* in ampicillin induced biofilm might explain the enhanced biofilm viability and biomass.

## Author Contributions

ZX and AW conceived of the study, participated in its design and coordination. YH, LY, and DC carried out the strains collection and samples preparation. LL and BL conducted the RNA extraction, library construction and RNA-seq. JL and BP performed the DEGs identification and annotation. JS, TS, JH, and MS carried out the bioinformatics analyses. JL, BZ, and YL performed the pathway analyses. All authors reviewed and approved the final manuscript.

## Conflict of Interest Statement

The authors declare that the research was conducted in the absence of any commercial or financial relationships that could be construed as a potential conflict of interest.

## Footnotes

1 faostat.fao.org

2 http://www.bioinformatics.babraham.ac.uk/projects/fastqc/

## Supplementary Material

Figure S1 The expression level of differentially expressed genes.

Table S1 Overview of the RNA-seq statistics.

Table S2 Differentially expressed genes of ampicillin-induced (A1, A2, and A3) and control (C1, C2, and C3) groups.

Table S3 GO enrichment analysis (Biological Process) of differentially expressed genes in ampicillin-induced (A1, A2, and A3) group compared to control (C1, C2, and C3).

Table S4 KEGG pathway enrichment analysis of differentially expressed genes in ampicillin-induced (A1, A2, and A3) group compared to control (C1, C2, and C3) group.
