# Supplementary figures and images for: Transcriptomics Study on Staphylococcus aureus Biofilm Under Low Concentration of Ampicillin
Source: Front Microbiol. 2018 Oct 30;9:2413. doi: 10.3389/fmicb.2018.02413 (PMC6218852; doi:10.3389/fmicb.2018.02413)

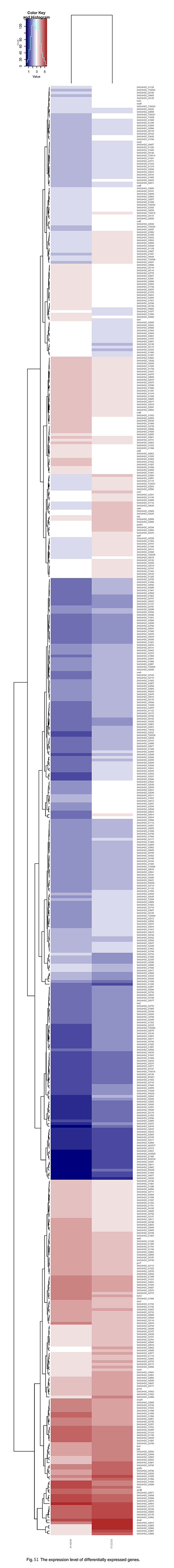

Supplement: Figure S1 — The expression level of differentially expressed genes. [file Image_1.TIF]
