# Supplementary material for: Transcriptomics Study on Staphylococcus aureus Biofilm Under Low Concentration of Ampicillin
Source: Front Microbiol. 2018 Oct 30;9:2413. doi: 10.3389/fmicb.2018.02413 (PMC6218852; doi:10.3389/fmicb.2018.02413)
Supplement: Table S1 — Overview of the RNA-seq statistics. [file Table_1.docx]

Table S1 Overview of the RNA-Seq statistics.

|  |  | A1 | | A2 | | A3 | | C1 | | C2 | | C3 | |  |
| --- | --- | --- | --- | --- | --- | --- | --- | --- | --- | --- | --- | --- | --- | --- |
|  |  | Read 1 | Read 2 | Read 1 | Read 2 | Read 1 | Read 2 | Read 1 | Read 2 | Read 1 | Read 2 | Read 1 | Read 2 | |
| Quality filter statistics | Original reads | 19,219,237 (100%) | 19,219,237 (100%) | 18,595,666 (100%) | 18,595,666 (100%) | 18,221,434 (100%) | 18,221,434 (100%) | 16,801,066 (100%) | 16,801,066 (100%) | 22,637,251 (100%) | 22,637,251 (100%) | 20,013,587 (100%) | 20,013,587 (100%) | |
|  | Adaptor contamination | 15,623 (0.08%) | 15,303 (0.08%) | 20,601 (0.11%) | 20,266 (0.11%) | 14,478 (0.08%) | 14,243 (0.08%) | 14,860 (0.09%) | 14,732 (0.09%) | 20,762 (0.09%) | 20,455 (0.09%) | 19,550 (0.10%) | 19,354 (0.10%) | |
|  | Low quality sequences | 2,146,199 (11.17%) | 899,563 (4.68%) | 1,970,809 (10.60%) | 308,541 (1.66%) | 1,726,034 (9.47%) | 277,254 (1.52%) | 1,850,952 (11.02%) | 285,729 (1.70%) | 2,112,511 (9.33%) | 342,146 (1.51%) | 2,181,149 (10.90%) | 333,419 (1.67%) | |
|  | Clean sequences | 16,723,117 (87.01%) | 16,723,117 (87.01%) | 16,501,790 (88.74%) | 16,501,790 (88.74%) | 16,383,442 (89.91%) | 16,383,442 (89.91%) | 14,842,294 (88.34%) | 14,842,294 (88.34%) | 20,378,466 (90.02%) | 20,378,466 (90.02%) | 17,700,874 (88.44%) | 17,700,874 (88.44%) | |
|  | Clean_Q20 | 97.38% | 96.29% | 99.07% | 96.57% | 99.14% | 96.8% | 99.11% | 96.45% | 99.16% | 96.82% | 99.11% | 96.49% | |
|  | Clean_Q30 | 91.6% | 89.13% | 96.81% | 89.91% | 96.99% | 90.46% | 96.91% | 89.59% | 97.05% | 90.54% | 96.9% | 89.77% | |
|  | Clean reads | 33,446,234 (87.01%) | | 33,003,580 (88.74%) | | 32,766,884 (89.91%) | | 29,684,588 (88.34%) | | 40,756,932 (90.02%) | | 35,401,748 (88.44%) | |  |
|  | Total bases | 5,765,771,100 (100%) | | 5,578,699,800 (100%) | | 5,466,430,200 (100%) | | 5,040,319,800 (100%) | | 6,791,175,300 (100%) | | 6,004,076,100 (100%) | |  |
|  | Clean bases | 4,841,432,296 (83.97%) | | 4,818,100,405 (86.37%) | | 4,788,164,625 (87.59%) | | 4,347,622,177 (86.26%) | | 5,934,460,615 (87.38%) | | 5,188,670,952 (86.42%) | |  |
| Mapped statistics | Mapped | 12,055,638 (36.04%) | 11,710,633 (35.01%) | 10,507,517 (31.84%) | 10,164,324 (30.8%) | 9,764,156 (29.8%) | 9,472,072 (28.91%) | 12,040,904 (40.56%) | 11,587,198 (39.03%) | 16,589,246 (40.7%) | 16,061,481 (39.41%) | 13,548,284 (38.27%) | 13,057,674 (36.88%) | |
|  | Total mapped | 23,766,271 (71.06%) | | 20,671,841 (62.64%) | | 19,236,228 (58.71%) | | 23,628,102 (79.6%) | | 32,650,727 (80.11%) | | 26,605,958 (75.15%) | |  |
|  | Multiple mapped | 30,417 (0.09%) | | 45,108 (0.14%) | | 31,651 (0.1%) | | 36,107 (0.12%) | | 51,120 (0.13%) | | 38,655 (0.11%) | |  |
|  | Uniquely mapped | 23,735,854 (70.97%) | | 20,626,733 (62.5%) | | 19,204,577 (58.61%) | | 23,591,995 (79.48%) | | 32,599,607 (79.99%) | | 26,567,303 (75.05%) | |  |
|  | Reads mapped to plus trand | 11,919,114 (35.64%) | | 10,366,302 (31.41%) | | 9,635,905 (29.41%) | | 11,858,800 (39.95%) | | 16,369,827 (40.16%) | | 13,358,654 (37.73%) | |  |
|  | Reads mapped to minus trand | 11,847,157 (35.42%) | | 10,305,539 (31.23%) | | 9,600,323 (29.3%) | | 11,769,302 (39.65%) | | 16,280,900 (39.95%) | | 13,247,304 (37.42%) | |  |
|  | Reads mapped in proper pairs | 11,206,457 (67.01%) | | 9,667,920 (58.59%) | | 9,063,431 (55.32%) | | 10,932,569 (73.66%) | | 15,317,661 (75.17%) | | 12,282,064 (69.39%) | |  |

Table 2 Statistics of DEGs

| DEGs | Normal state versus mid-term state | Normal state versus VPNC state | Mid-term state versus VPNC state | Initial phase | Latter phase | Whole process | Type I | Type II | Type III |
| --- | --- | --- | --- | --- | --- | --- | --- | --- | --- |
| BM-LA14526 | 122 | 109 | 173 | 32 | 53 | 11 | 21 | 42 | 11 |
| BM-LA14527 | 261 | 303 | 348 | 77 | 143 | 8 | 69 | 135 | 8 |
| BM-LA14528 | 107 | 109 | 7 | 82 | 1 | 0 | 82 | 1 | 0 |

DEGs in the initial phase: common DEGs in normal state versus mid-term state and normal state versus VPNC state groups; DEGs in the latter phase: common DEGs in mid-term state versus VPNC state and normal state versus VPNC state groups; DEGs in the whole process: DEGs both in the initial and latter phase.
